# Recognition of Anemia in Elderly People in a Rural Community Hospital

**DOI:** 10.3390/ijerph182111179

**Published:** 2021-10-25

**Authors:** Shiho Amano, Ryuichi Ohta, Chiaki Sano

**Affiliations:** 1 Community Care, Unnan City Hospital, Daito-cho Iida, Unnan 699-1221, Japan; amano_shiho95@yahoo.co.jp; 2 Department of Community Medicine Management, Faculty of Medicine, Shimane University, Enya-cho, Izumo 693-8501, Japan; sanochi@med.shimane-u.ac.jp

**Keywords:** anemia, recognition, elderly patients, ageism, Japan, rural

## Abstract

Anemia in the elderly is a common disease associated with increased mortality and hospitalization rates. It is not clear how adequately elderly patients are assessed and treated in actual clinical practice. This study clarifies the frequency of anemia recognition before assessment and the factors related to recognition among older people in a rural community hospital. This cross-sectional study evaluated 156 elderly patients aged > 65 years. Data on several different variables were collected from patient medical records. Anemia was defined as a hemoglobin level < 11 g/dL. Patients were classified into “anemia recognition” and “no anemia recognition” groups. Statistical analysis of the data included multivariable logistic regression to examine the association between anemia recognition and other factors. The anemia recognition group comprised 63 (40.4%) patients. Age was significantly associated with the recognition of anemia (adjusted odds ratio = 0.70, 95% confidence interval: 0.53–0.92, *p* = 0.011). Appropriate medical care should be provided to the elderly; however, it may be limited according to age.

## 1. Introduction

Anemia in the elderly is a common disease that is associated with increased mortality and hospitalization rates. The management of anemia in the elderly is critical, and appropriate assessment and consultation with hematologists are essential. According to the World Health Organization (WHO), anemia is defined as a hemoglobin (Hb) level below 13 g/dL in men and 12 g/dL in women [1]. The prevalence of anemia is 17% in elderly people (>65 years old), 40% in hospitalized patients, and 12% in the community [2]. The prevalence of anemia increases with age [3]. As the appropriate definition of anemia in elderly people is unclear, the lower limit of the normal range of Hb in healthy elderly people is not very different from that in younger generations [4]. Anemia in elderly people has been reported to be related to mortality, hospitalization rate, dementia, falls, loss of physical function, and deterioration in quality of life (QOL) [3]. In a cohort study of 17,030 people aged 66 years and older, an inverse J-shaped relationship between Hb levels and all-cause mortality and the hospitalization rate for cardiovascular events was reported; the lowest risk for mortality occurred at Hb values between 13 and 15 g/dL for women and 14–17 g/dL for men, with or without chronic kidney disease (CKD) [3,5]. As the standard definition of anemia among older people is not different from that of younger generations, anemia in the elderly should be managed in the same way as in younger people.

The causes of anemia in elderly people can be divided into three groups: malnutrition, anemia of chronic disease, and anemia of unknown cause. Malnutrition is treatable and relates to deficiencies in iron, vitamin B12, and folic acid [3]. The most common cause of anemia is iron-deficiency [6]. The common risk factors in elderly people include chronic alcohol use, malnutrition, CKD, liver diseases, myelodysplastic disorders, gastrointestinal bleeding, cancer, androgen deficiency, and age-related decline in stem cell proliferation [2]. The appropriate definition of anemia in elderly people is not significantly different from that in young people; nonetheless, mild anemia seems to be disregarded as “age-related.” Considering the increase in mortality and hospitalization rates associated with anemia, any evidence of anemia in the elderly should be investigated. However, it is thought that elderly people should not be actively investigated in consideration of invasiveness and limited longevity, particularly in both older people and caregivers in rural areas [7]. Healthy elderly individuals can tolerate the invasion associated with investigations and tests, and those residing in rural areas may exhibit improvements in their physical and cognitive functions [8,9]. Therefore, older people should be intensively investigated with a view to improving their health.

Mortality and hospitalization rates in the elderly increase even in cases of mild anemia, but it is not clear how adequately patients are assessed and treated in actual clinical practice. In particular, in rural areas, an increasing number of older people require medical care, including for an increased incidence of anemia in rural community hospitals. The demand for medical care among older patients imposes a heavy burden on rural medical staff [10,11]. The assessment and treatment of anemia in older adults are important, as these can mitigate the symptoms in older adults and reduce the number of patients requiring complicated medical care. Considering the aging societies in Japan and worldwide, the issue of anemia in the elderly should be investigated in order to improve the future of older people’s lives. Nonetheless, no study has clarified whether an improvement in anemia improves mortality and hospitalization rates. The deterioration in prognosis due to anemia is clear, and assessment of the causes of anemia may improve the prognosis; this may contribute to the improvement in return-to-home rates and QOL of elderly patients with advanced frailty. It is necessary to recognize the patient’s anemia before conducting an assessment to identify the cause of the anemia. The assessment may include laboratory tests, gastrointestinal endoscopy, and fecal occult blood testing. Therefore, this study aimed to clarify the frequency of recognition of anemia and the factors related to recognition among older people in a rural community hospital.

## 2. Materials and Methods

### 2.1. Setting

The Unnan City Hospital is a rural community hospital located in the southeastern part of Shimane Prefecture, Japan. It is the only public hospital in Unnan City. The hospital staff comprises 27 physicians, 197 nurses, 7 pharmacists, 15 clinical technicians, 37 therapists (22 physical therapists, 12 occupational therapists, and 3 speech therapists), 4 nutritionists, and 34 clerks. There was no other medical institution with a recovery rehabilitation unit in Unnan City [12]. In 2021, Unnan City had a total population of 37,039 (17,869 men and 19,170 women). The proportion of people aged > 65 years was 39.46% [13]. 

### 2.2. Participants

The study participants were selected from patients admitted to the Department of General Medicine of Unnan City Hospital. Patients aged > 65 years with anemia on initial laboratory tests were included in the study. Patients were excluded if admitted to other departments or hospitalized for medical checkups. Patient data were retrospectively collected between April 2020 and December 2020 from the electronic medical records of the hospital. Cases with missing data were excluded from the analysis.

### 2.3. Sample Size

Regarding the sample size calculation, 126 participants were needed for an 80% statistical power and a 5% α error to detect a difference in the average age of 5 years (standard deviation [SD] of 10) between the anemia recognition and no anemia recognition groups. 

### 2.4. Data Collection

The presence of anemia recognition was considered as a dependent variable in this study. The lower limit of the normal range of Hb in healthy older patients is not clearly defined. The average Hb level decreases with age in two different databases (NHANES-III and Scripps-Kaiser) [4]. One study of healthy men and women aged 70–88 years in Sweden showed that a lower limit of the normal Hb range of 11.5 g/dL was appropriate for those aged 80–82 years [14]. A Hb level of 11.0 g/dL is frequently used as the lower limit in Japanese elderly people [15]. As the average age of participants in this study was 87.6 years, anemia was defined as a Hb level of <11.0 g/dL. 

The risk factors for anemia were based on previous research and were evaluated as independent variables [3,5]. The data for these variables were also collected from electronic medical records and included age; sex; albumin level; body mass index (BMI); dependent conditions; the Charlson comorbidity index (CCI) [16], which was calculated based on the presence of myocardial infarction, heart failure, brain hemorrhage, brain stroke, dementia, chronic obstructive pulmonary disease, connective tissue disease, peptic ulcer disease, hepatic cirrhosis, diabetes, hemiplegia, CKD, estimated glomerular filtration rate and malignancy; the functional independence measure (FIM) [17]; presence of anemia; and presence of dysphagia. Data regarding the diagnosis at admission and results of laboratory test conducted within 1 week after admission (albumin levels, Hb levels, and the estimated glomerular filtration rate) were collected from the medical records. Patients were classified into “anemia recognition” and “no anemia recognition” groups by referring to the medical records within one week after admission. We judged the “recognition of anemia” by whether “anemia” was listed in patients’ medical records by the attending physicians. The FIM was collected from the data at admission. Any case of an intervention from a speech-language-hearing therapist was defined as dysphagia.

### 2.5. Statistical Analysis

For continuous variables, the normality of the data was tested before applying the statistical tests. Parametric and nonparametric data were analyzed using the t-test and Mann-Whitney U test, respectively. Nominal variables were analyzed using the Fisher’s exact test. The following continuous variables were dichotomized for analysis as binomial variables: CCI (>5 and <5) [16] and dependent condition (dependence ≥ 1 and 0) [12]. Age was categorized every 5 years for analysis considering the availability in clinical settings. Multivariate logistic regression analysis was conducted to examine the association between the presence of anemia recognition and other factors. Variables associated with anemia recognition in the univariate regression model were used in the multivariate logistic regression model. All data analyses were performed using Easy R (version 1.54; R Foundation for Statistical Computing, Vienna, Austria). Statistical significance was defined as *p* < 0.05 [18].

### 2.6. Ethical Considerations

The anonymity and confidentiality of patient information were ensured. The patients and their families were informed about using their clinical data for the performance and publication of this study, and informed consent was obtained. The research information was posted on the hospital’s website without any patient details. The contact information of the hospital representative was listed on the website, such that it was possible to answer any questions about this research at any time. All procedures in this study were performed in compliance with the Declaration of Helsinki and its later amendments. The Clinical Ethics Committee of Unnan City Hospital approved the study protocol (approval number: 20210005 (the date of approval: 18 May 2021)).

## 3. Results

### 3.1. Demographics of the Participants

**Figure 1:**
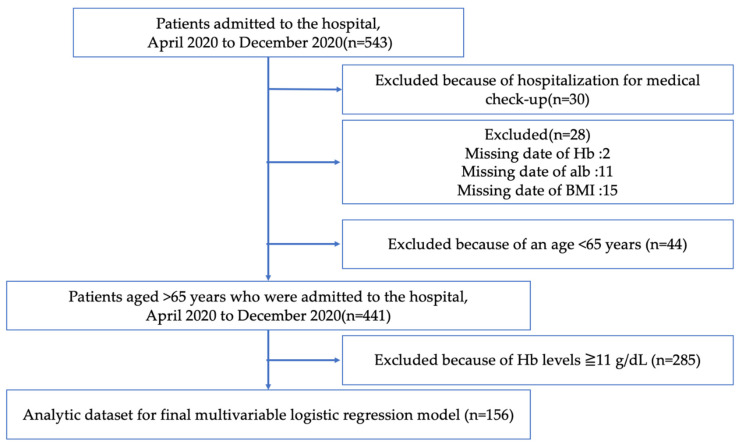
Flowchart of the study population selection process.

Figure 1 shows the flowchart of the study population selection process. From April to December 2020, 543 patients were admitted to the Department of General Medicine of Unnan City Hospital. A total of 30, 28, and 44 patients were excluded because of hospitalization for medical checkup, lack of data (no Hb data: 2, no albumin data: 11, no BMI data: 15), and an age of <65 years, respectively.

The prevalence of anemia was 35.4% (156 out of 441 patients). Hence, a total of 156 patients with anemia (Hb < 11 g/dL) were included in this study (Figure 1), of which 63 (40.4%) were categorized as belonging to the anemia recognition group. The average age of the patients was 84.81 years (standard deviation (SD) = 7.71) and 89.49 years (SD = 6.30) in the “anemia recognition” and “no anemia recognition” groups, respectively. Age, BMI, Hb levels, and the prevalence of peptic ulcer disease, FIM, and dysphasia differed significantly between the two groups (Table 1). The most common diagnoses at admission were heart failure, followed by urinary tract infection, aspiration pneumonia, and gastrointestinal bleeding (Table 2).

### 3.2. Association between the Recognition of Anemia and Influential Factors

Age, albumin, BMI, a CCI > 5, dysphagia, and FIM were analyzed using multivariate logistic regression. Younger age was significantly associated with more frequent recognition of anemia (adjusted odds ratio = 0.70, 95% confidence interval: 0.53–0.92, *p* = 0.011). Albumin, BMI, dysphagia, and FIM showed no significant association with anemia recognition (Table 3).

## 4. Discussion

This study revealed factors associated with anemia recognition among elderly people in a rural community hospital and showed that the recognition of anemia before conducting an assessment occurred in less than half of the patients. The prevalence of anemia was 35.4%, similar to that in a previous study that reported that the prevalence of anemia was 40% in hospitalized patients [2]. However, among the patients with anemia, the proportion with anemia recognition was 40.4%. Age was the only factor that showed a significant association with anemia recognition in the multivariate logistic regression. 

Since age was the only factor associated with anemia recognition, it suggests that ageism may exist in recognizing anemia in the elderly. Ageism is an age-based stereotype, prejudice, and discrimination against the elderly, and previous studies have shown a strong association between ageism and the physical and psychological health risks of the elderly [19,20,21]. The speed of aging varies greatly among individuals, and the differences between individuals tend to increase with age [22,23]. In addition, physical, mental, and social consequences of aging vary from person to person [24]. Elderly people often have multiple comorbidities in addition to aging, and there is no clear standard as to how much medical assessment should be performed; it is largely left to each clinician to decide [25]. Based on previous studies, there is a possibility that the provision of medical care, such as clinical assessment and intervention, may be restricted because of age and clinicians’ perceptions of deterioration in the physical and mental functions of older patients, without solid evidence [20,26]. We also measured comorbidities (CCI), dependence, nutritional status (BMI, albumin), nursing home residents, and dementia as factors other than age, but age was the only factor that showed a significant association with anemia recognition by multivariate logistic regression. Therefore, age may be related to the recognition of anemia in the elderly. Factors related to prognosis in the elderly are diverse. It is possible that age is not the only factor defining prognosis and the rate of discharge to home, and may be associated with rehabilitation and social factors in the elderly [27]. Appropriate assessment and treatment based on the recognition of anemia may improve the prognosis of elderly people. Therefore, it is necessary to provide appropriate medical care to the elderly, considering the presence of ageism.

This study indicates that medical care for the elderly may be affected by ageism, as there are individual differences in aging. This study reveals that physicians may more often recognize anemia as a clinical problem in younger people than in older people. This study suggests that age may be related to poor recognition of anemia in the elderly, showing the trend of ageism among physicians. For better medical care, elderly patients should be treated based on the following three factors: comorbidities, dependence, and nutritional status. Ageism can be caused by patients and caregivers because social reputations can influence their perception of aging [28]. In addition, caregivers’ stress in home care can induce ageism due to depression and the desire to avoid taking care of older people [29]. As mentioned initially, the causes of anemia in older adults can be divided into three groups: malnutrition, anemia of chronic disease, and anemia of unknown cause. Malnutrition is treatable and includes deficiencies in iron, vitamin B12, and folic acid. Anemia can be assessed with a relatively less-invasive blood test. Therefore, anemia may result in increased medical care if we do not assess it because of age. In addition, the Hb levels of the patients were 8.73 g/dL (SD = 1.53) and 9.76 g/dL (SD = 1.16) in the anemia recognition and no anemia recognition groups, respectively. This indicates that mild anemia was less commonly recognized. Previous studies have shown that even minor anemia is associated with poor prognosis [30,31]. Therefore, clinicians should be careful in the recognition, assessment, and treatment of minor anemia. It is clear that mortality and hospitalization rates in the elderly will increase even if anemia is mild, but it is not clear whether assessment and treatment through appropriate recognition can improve outcomes. Future studies may reveal that anemia assessment in the elderly is associated with improved prognosis and changes in QOL. 

This study has some limitations. The sample size was small because of the limited study period. Further studies with a larger sample size are required because the number of cases may be relatively small, the detection rate may be low, and factors other than age may also be related to lack of recognition of anemia. We selected all patients who were admitted to the Department of General Medicine of our hospital during the 8-month study period in order to avoid selection bias. Another limitation is that it was a single-center study, which may affect the external validity. On the other hand, aging of the population is progressing in this community, and this represents one of the typical social structures of the aging society that not only Japan but also the world will encounter in the future. This study represents this population in rural community hospitals because all patients were evaluated by general practitioners with no specialty biases. A further limitation is that consideration is needed for unknown variables regarding social factors associated with anemia recognition. In addition, since anemia recognition and comorbidities (CCI) were confirmed by referring to the medical records, accurate data may not have been extracted because of omissions in the medical records. Thus, there is a possibility that some patients in whom anemia was recognized were assigned to the “no anemia recognition group” because recognition of anemia was not documented in their medical records.

## 5. Conclusions

The proportion of patients with anemia recognition in the elderly was 40.4% in this rural community hospital. Age was the only factor that showed a significant association with anemia recognition. Appropriate medical care should be provided to the elderly, but may be limited by age. It is clear that mortality and hospitalization rates in the elderly will increase with anemia, but it needs to be clarified whether assessment and treatment can improve outcomes.

## Figures and Tables

**Table 1:** Characteristics of the study participants (n = 156).

	Anemia Recognition		
Characteristics	Negative (n = 93)	Positive (n = 63)	*p*-Value
Age (years), mean (SD) 65–69, n (%) 70–74, n (%) 75–79, n (%) 80–84, n (%) 85–89, n (%) 90–94, n (%) 95–99, n (%)	89.49 (6.30) 0 (0.00) 2 (2.15) 5 (5.38) 14 (15.05) 22 (23.66) 25 (26.88) 25 (26.88)	84.81 (7.71) 3 (4.76) 7 (11.11) 5 (7.94) 7 (11.11) 24 (38.10) 14 (22.22) 3 (4.76)	<0.001
Sex, male, n (%)	34 (36.6)	22 (34.9)	0.866
Albumin (g/dL), mean (SD)	3.15 (0.60)	3.13 (0.62)	0.867
BMI (kg/m^2^), mean (SD)	19.15 (3.00)	20.89 (5.17)	0.009
Hb (g/dL), mean (SD)	9.76 (1.16)	8.73 (1.53)	<0.001
Dependent condition, n (%) (dependence ≥ 1)	61 (65.6)	32 (50.8)	0.070
CCI score (≥5), n (%)	71 (76.3)	45 (71.4)	0.576
Myocardial infarction, n (%)	7 (7.5)	1 (1.6)	0.144
Congestive heart failure, n (%)	22 (23.7)	18 (28.6)	0.576
Brain hemorrhage, n (%)	11 (11.8)	4 (6.3)	0.285
Brain stroke, n (%)	18 (19.4)	11 (17.5)	0.836
Dementia, n (%)	24 (25.8)	9 (14.3)	0.110
Chronic obstructive pulmonary disease, n (%)	6 (6.5)	5 (7.9)	0.757
Connective tissue disease, n (%)	6 (6.5)	2 (3.2)	0.475
Peptic ulcer disease, n (%)	5 (5.4)	16 (25.4)	<0.001
Hepatic cirrhosis, n (%)	2 (2.2)	1 (1.6)	1
Diabetes, n (%)	17 (18.3)	5 (12.7)	0.383
Hemiplegia, n (%)	1 (1.1)	3 (4.8)	0.304
Chronic kidney disease, n (%)	61 (65.6)	50 (79.4)	0.073
Malignancy, n (%)	21 (22.6)	14 (22.2)	1
Discharge to facility, n (%)	21 (23.9)	13 (22)	0.844
Admission from facility, n (%)	16 (17.2)	10 (15.9)	1
Dysphagia, n (%)	23 (25)	5 (7.9)	<0.001
FIM at admission, mean (SD)	61.88 (39.69)	79.81 (43.47)	0.017

SD = standard deviation, BMI = body mass index, CCI = Charlson comorbidity index, eGFR = estimated glomerular filtration rate, FIM = functional independence measure, Hb = hemoglobin.

**Table 2:** Participants’ diagnoses on admission (n = 156).

Number	Disease	Number of Cases	%	Number	Disease	Number of Cases	%
1	Heart failure	21	13.5	12	Brain hemorrhage	2	1.3
2	UTI	18	11.5	13	Compression fracture	2	1.3
3	Aspiration pneumonia	13	8.3	14	Dehydration	2	1.3
4	Gastrointestinal bleeding	9	5.8	15	Hypoglycemia	2	1.3
5	Brain stroke	8	5.1	16	RS3PE	2	1.3
6	Pneumonia	8	5.1	17	TIA	2	1.3
7	Cancer	7	4.5	18	Sepsis	2	1.3
8	Pseudogout	6	3.8	19	CD colitis	2	1.3
9	Syncope	6	3.8	20	Myocardial infarction	2	1.3
10	Cellulitis	4	2.6	21	Other	34	21.8
11	Anemia	4	2.6				

UTI = urinary tract infection, RS3PE = remitting seronegative symmetrical synovitis with pitting edema, TIA = transit ischemic attack, CD = *Clostridium difficile.*

**Table 3:** Association between anemia recognition and influencing factors.

Factor	Odds Ratio	95% CI	*p*-Value
Age (every 5 years)	0.70	0.53–0.92	0.011
Albumin	0.80	0.45–1.44	0.461
BMI	1.07	0.97–1.19	0.178
CCI ≥ 5	0.69	0.31–1.54	0.361
Dysphagia	0.40	0.13–1.20	0.103
FIM	1.00	0.99–1.01	0.509

CI = confidence interval, BMI = body mass index, CCI = Charlson comorbidity index, FIM = functional independence measure.

## Data Availability

The data that support the findings of this study are available from the corresponding author, S. Amano, upon reasonable request.
